# Improving the microbiological diagnosis of tuberculous meningitis: A prospective, international, multicentre comparison of conventional and modified Ziehl–Neelsen stain, GeneXpert, and culture of cerebrospinal fluid

**DOI:** 10.1016/j.jinf.2018.09.003

**Published:** 2018-12

**Authors:** A. Dorothee Heemskerk, Joseph Donovan, Do Dang Anh Thu, Suzaan Marais, Lidya Chaidir, Vu Thi Mong Dung, Chad M. Centner, Vu Thi Ngoc Ha, Jessi Annisa, Sofiati Dian, Louise Bovijn, Nguyen Thi Hoang Mai, Nguyen Hoan Phu, Nguyen Van Vinh Chau, Ahmad Rizal Ganiem, Cao Thao Van, Ronald B. Geskus, Nguyen Thuy Thuong Thuong, Rovina Ruslami, Graeme Meintjes, Reinout van Crevel, Robert J. Wilkinson, Guy E. Thwaites

**Affiliations:** a Oxford University Clinical Research Unit, Ho Chi Minh City, Vietnam; b Centre for Tropical Medicine and Global Health, Nuffield Department of Medicine, University of Oxford, Oxford, United Kingdom; c Wellcome Center for Infectious Diseases Research in Africa and Department of Medicine, University of Cape Town, Observatory 7925, South Africa; d Department of Neurology, Inkosi Albert Luthuli Central Hospital and University of KwaZulu-Natal, Durban, South Africa; e Infectious Disease Research Center, Faculty of Medicine, Universitas Padjadjaran/Hasan Sadikin Hospital, Bandung, Indonesia; f Division of Medical Microbiology, University of Cape Town and National Health Laboratory Service, Groote Schuur Hospital, Cape Town, South Africa; g Department of Medicine and Radboud Center for Infectious Diseases, Radboud University Medical Center, Nijmegen, Netherlands; h Hospital for Tropical Diseases, Ho Chi Minh City, Vietnam; i Department of Medicine, Imperial College London, W2 1PG, United Kingdom; j Francis Crick Institute, NW1 1AT, United Kingdom

**Keywords:** Tuberculous meningitis, Diagnosis, Ziehl–Neelsen stain, Cytospin, Xpert MTB/RIF

## Abstract

**Objectives:**

Tuberculous meningitis (TBM) is the severest form of tuberculosis, but current diagnostic tests are insensitive. Recent reports suggest simple modifications to conventional cerebrospinal fluid (CSF) Ziehl–Neelsen (ZN) staining may greatly improve sensitivity. We sought to define the performance of modified and conventional ZN stain for TBM diagnosis.

**Methods:**

In hospitals in Vietnam, South Africa and Indonesia we conducted a prospective study of modified ZN with or without cytospin, conventional ZN smear, GeneXpert, and culture on CSF in adults with suspected TBM.

**Results:**

A total of 618 individuals were enrolled across 3 sites. Compared with the TBM clinical diagnostic gold standard for research (definite probable or possible TBM), sensitivity of conventional ZN and modified ZN with cytospin were 33.9% and 34.5% respectively (*p* = 1.0 for the difference between tests), compared with culture 31.8% and Xpert 25.1%. Using culture as a reference, sensitivities of conventional ZN, modified ZN with cytospin, and Xpert were 66.4%, 67.5%, and 72.3%, respectively. Higher CSF volume and lactate, and lower CSF:blood glucose ratio were independently associated with microbiologically confirmed TBM.

**Conclusions:**

Modified ZN stain does not improve diagnosis of TBM. Currently available tests are insensitive, but testing large CSF volumes improves performance. New diagnostic tests for TBM are urgently required.

## Highlights

• Modified ZN staining of CSF with a cytospin step was not superior to conventional ZN staining for the diagnosis of TBM.
• Modified ZN staining of CSF without a cytospin step was inferior to conventional ZN staining for the diagnosis of TBM.
• Higher CSF volume and lactate, and lower blood glucose ratio were independently associated with microbiological confirmation of TBM.

## Introduction

Tuberculous meningitis (TBM) is the most devastating form of infection with Mycobacterium tuberculosis (MTB). Over half of treated TBM patients die or suffer severe neurological sequelae,^1^ largely due to late diagnosis. For HIV co-infected patients TBM mortality is around 60%.^2^, ^3^ Worldwide, smear microscopy on cerebrospinal fluid (CSF) following conventional Ziehl–Neelsen staining (CZN) is insensitive. Culture of MTB takes at least 2 weeks and is therefore too slow to be clinically relevant during the acute phase. Xpert MTB/RIF® (Cepheid) has shown improved performance over ZN staining in most centres. However, cartridges are expensive and the test still lacks sensitivity (around 60% against a clinical gold standard).^4^ New highly sensitive, affordable diagnostic methods are urgently required to improve TBM outcomes.

Chen et al. devised a modification to the CZN stain intended to enhance intracellular staining of mycobacteria in CSF.^5^ The modified technique involves two additional steps to CZN; a cytospin step and an additional Triton-X processing step (MZN) (described in supplementary material 1). The principle is staining of intracellular bacilli, maintaining cell architecture by cytospinning and permeabilising cells with Triton-X for intracellular access of carbolfuchsin-dye. These simple modifications are achievable in most settings. In a pilot study, including 29 patients with culture confirmed TBM (and 48 samples of CSF); all samples (100%) stained positive using the MZN, compared to only 8 samples (27.6%) positive by CZN stain. In the MZN smears, leucocyte integrity was maintained and both intracellular and extracellular staining of mycobacteria was enhanced.^5^

A larger study then assessed the method in 280 patients with a clinical diagnosis of TBM, and the findings affirmed the pilot study results. Against a clinical diagnostic gold standard the sensitivity of CZN on CSF was 3.3% (95% confidence intervals (CI) 1.6–6.7%), compared to 82.9% (95% CI 77.4–87.3%) for the MZN stain. However, acid-fast bacilli (AFB) were also seen in six patients with cryptococcal (*n* = 4) and pyogenic bacterial (*n* = 2) meningitis, yielding a diagnostic specificity of 85.0%.^6^ A later study on individuals from the same geographical area showed improved sensitivity of the MZN on CSF when compared to Xpert against a clinical diagnosis as a gold standard (88.5% vs. 36.5%), but greatly reduced specificity (71.4% vs. 100.0%).^7^

The objective of the current study was to evaluate the performance of MZN in TBM patients in three different clinical settings in Vietnam, South Africa and Indonesia, against CZN smear, mycobacterial culture and Xpert, using a clinical case definition for research as a gold standard.^8^ The second objective was to investigate the clinical predictors of CSF microbiological confirmation of TBM.

## Methods

### Study design and participants

This was a prospective, multicentre comparison of diagnostic tests for TBM. Patients were screened for eligibility at three different study sites; The Hospital for Tropical Diseases, Ho Chi Minh City, Vietnam; GF Jooste Hospital and Mitchell's Plain Hospital, Cape Town, South Africa; and Hasan Sadikin Hospital, Bandung, Indonesia. Adults (≥18 years) who were offered lumbar puncture as a part of routine care for suspected brain infection were eligible for enrolment. Patients were excluded if bacterial meningitis was suspected (cloudy or pus-like CSF; as these patients are usually clinically distinguished from those with suspected TBM), lumbar puncture was contraindicated, or if no informed consent was obtained. In South Africa patients with impaired consciousness were enrolled, and patient consent was sought when capacity was regained. If death occurred before capacity was regained data was included, following ethical approval. The study was approved by all local and national ethical review boards: The Hospital for Tropical Diseases, Ho Chi Minh City, Vietnam (approval number 134/BB-HDDD), Oxford Tropical Research Ethics Committee (OxTREC) (approval number 51-14), University of Cape Town Faculty of Health Sciences Human Research Ethics Committee (approval number HREC REF: 730/2014), Human Research Ethics Committee of Faculty of Medicine Universitas Padjadjaran Bandung (approval number: 299/UN6.C2.12/KEPK/PN/2014).

### Laboratory methods for mycobacterial detection

At least 3 ml of CSF was used for mycobacterial targeted diagnostic tests where this volume was available. However in 14 patients <3 ml of CSF was available. Laboratory technicians were blinded to the suspected diagnosis. Prior to centrifuging, 0.5 ml of uncentrifuged CSF was aliquoted for modified ZN smear with cytospin preparation of the slide (MZN with cytospin). The remainder of the CSF sample was centrifuged at 3000 g for 15 min. The supernatant was removed, leaving approximately 500 µl in which the deposit was re-suspended. In Vietnam and Indonesia 100 µl was used for CZN, 100 µl was submitted for mycobacterial liquid culture and 200 µl was used for GeneXpert MTB/RIF testing (Xpert). Additionally, 100 µl of the deposit was used to perform MZN without cytospin, to assess the added value of cytospin which may be unavailable in some settings. In South Africa equal CSF deposit proportions were used for CZN, MZN without cytospin, liquid culture and Xpert. MZN with and without cytospin were performed as outlined in supplementary material 1.^5^, ^6^ Slide reading was pragmatic, and followed the standard procedure of each site. In Vietnam, slides were read until AFB were identified, and then 100 fields were read: if no positive fields were identified the entire slide was read. In South Africa the first 100 fields of the slide were read. In Indonesia the entire slide was read. Slide reading methods were consistent across all ZN variations within a site. Mycobacterial liquid culture was performed using BACTEC mycobacteria growth indicator tube (MGIT) (Becton Dickinson) in Vietnam and South Africa, and microscopic observation drug susceptibility assay (MODS) in Indonesia. CZN, Xpert and culture were performed according to standard procedures.^4^

### Statistical analysis

Sample size calculations were performed prior to recruitment, aiming at detecting an increase in sensitivity of the MZN, compared to CZN staining (+10% (power 79%), +30% (power 87%) and +20% (power 79%)) for Vietnam, South Africa and Indonesia respectively; translating to 120, 50 and 82 patients with TBM for each site. For the analysis only baseline lumbar punctures were used (where ‘baseline’ was defined as a lumbar puncture performed +/−10 days of enrolment). The published diagnostic gold standard for research was used to define the diagnostic likelihood of TBM (supplementary Table 1).^8^ In South Africa a modification of the clinical diagnostic score was used: if patients did not receive any antitubercular treatment, and remained alive at 6-month follow-up, they were re-assigned to not-TBM, regardless of their original diagnostic score. Patients were excluded from the analysis of a diagnostic test's performance if results were missing for that test. The sensitivity, specificity, positive predictive value (PPV) and negative predictive value (NPV) (with associated 95% Wilson CIs) were calculated for CZN, MZN with cytospin, culture, and Xpert, against the clinical gold standard for TBM diagnosis, as well as for non-culture tests against culture as a reference. The sensitivity and specificity of MZN with cytospin and MZN without cytospin were compared with each other against the clinical gold standard (based on the baseline CSF sample) using McNemar's test. Logistic regression was used to identify clinical and laboratory factors associated with microbiological diagnosis (defined as positive CZN, Xpert or culture) of TBM. Statistical analysis was performed using the programming language R (version 3.5.0).

## Results

**Fig. 1:**
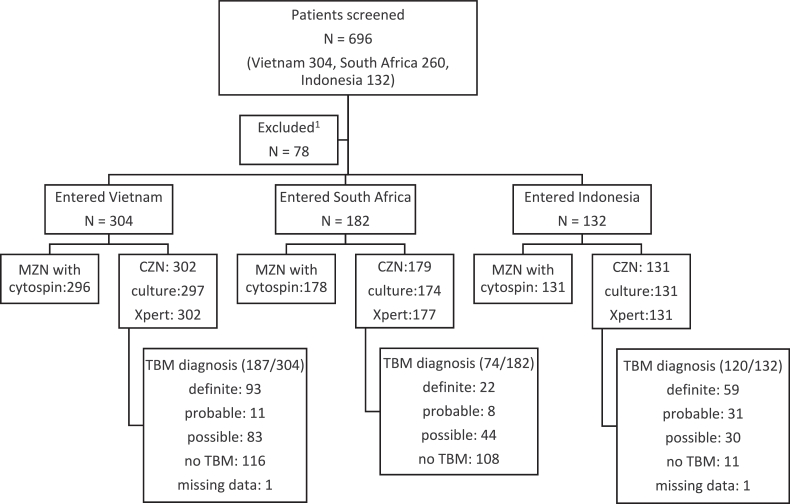
Flow chart of patients screened and recruited to study. ZN; Ziehl–Neelsen smear, MZN; modified Ziehl–Neelsen smear, CZN; conventional ZN smear, culture; mycobacterial culture by MGIT (Becton Dickinson, USA) (Vietnam and South Africa) or MODS (Indonesia), Xpert; Xpert MTB/RIF (Cepheid), TBM; tuberculous meningitis. ^1^Reasons for exclusion were as follows; entry criteria not met (*n* = 18), at least one exclusion criteria met (*n* = 50), consent not given by patient (*n* = 9), consent could not be obtained (*n* = 1).

A total of 618 patients were consecutively enrolled between January 2015 and September 2016, as shown in Fig. 1; 304 in Vietnam, 182 in South Africa and 132 in Indonesia. Clinical diagnostic scores for TBM were; 174 (28.2%) definite, 50 (8.1%) probable, 157 (25.4%) possible, and 235 (38.1%) not-TBM. There was insufficient data to calculate the clinical diagnostic score in 2 (0.3%) cases, and to identify a baseline lumbar puncture in 3 (0.5%) cases. Baseline characteristics are shown stratified by site in Table 1 and stratified by TBM diagnosis in supplementary Table 2.

**Table 1:** Baseline characteristics stratified by site.

	Total no^a^	Vietnam (*N* = 304)	Total no^a^	South Africa (*N* = 182)	Total no^a^	Indonesia (*N* = 132)	Total no^a^	Total (*N* = 618)
**Age (years)** (Median(IQR))	304	40 (30, 53)	182	39 (31, 49)	132	30 (22, 39)	618	37 (28, 50)
**Sex (male)** (No (%))	304	184 (60.5%)	182	91 (50.0%)	132	79 (59.8%)	618	354 (57.3%)
**Final diagnosis** (No (%))	303		182		131		616	
Definite TBM		93 (30.6%)		22 (12.1%)		59 (44.7%)		174 (28.2%)
Probable TBM		11 (3.6%)		8 (4.4%)		31 (23.5%)		50 (8.1%)
Possible TBM		83 (27.3%)		44 (24.2%)		30 (22.7%)		157 (25.4%)
Not TBM		116 (38.2%)		108 (59.3%)		11 (8.3%)		235 (38.0%)
**HIV status** (No (%))	304		182		132		618	
Positive		23 (7.6%)		151 (83.0%)		20 (15.2%)		194 (31.4%)
Negative		185 (60.9%)		31 (17.0%)		110 (83.3%)		326 (52.8%)
Unknown		96 (31.6%)		0		2 (1.5%)		98 (15.9%)
								
**Duration of illness (days)** (Median(IQR))	300	9 (4, 14)	172	7 (3, 14)	129	14 (7, 30)	601	9 (5, 16)
**Known history of TB** (No (%))	265	17 (5.6%)	173	86 (47.3%)	132	41 (31.1%)	570	144 (23.3%)
**TBM MRC Grade**^b^ (No (%))	187		74		119		380	
1		64 (34.2%)		36 (48.6%)		6 (5.0%)		106 (27.9%)
2		90 (48.1%)		37 (50.0%)		93 (78.2%)		220 (57.9%)
3		33 (17.6%)		1 (1.4%)		20 (16.8%)		54 (14.2%)
**Cranial nerve palsy** (No (%))	303	41 (13.5%)	182	8 (4.4%)	132	84 (63.6%)	617	133 (21.5%)
**Hemiplegia** (No (%))	297	23 (7.6%)	182	9 (4.9%)	132	42 (31.8%)	611	74 (11.9%)
**Paraplegia** (No (%))	298	8 (2.6%)	182	1 (0.5%)	132	19 (14.4%)	612	28 (4.5%)
**Tetraplegia** (No (%))	294	1 (0.3%)	182	0 (0.0%)	132	5 (3.8%)	608	6 (1.0%)
**Seizures** (No (%))	293	39 (12.8%)	178	19 (10.4%)	132	16 (12.1%)	603	74 (12.0%)
**GCS** (Median(IQR))	303	13 (11, 15)	182	15 (14, 15)	131	13 (12, 15)	616	14 (12, 15)
**Chest X-ray** (No (%))	293		153		129		575	
Pulmonary TB		45 (14.8%)		57 (31.3%)		70 (53.0%)		172 (27.8%)
Miliary TB		19 (6.3%)		5 (2.7%)		12 (9.0%)		36 (5.8%)
Normal		180 (59.2%)		68 (37.4%)		33 (25.0%)		281 (45.5%)
Other^c^		49 (16.1%)		23 (12.6%)		14 (10.6%)		86 (13.9%)
**CSF WCC (per mm3)** (Median(IQR))	300	164 (30, 439)	180	1.5 (0, 61)	132	117 (6, 321)	612	80 (3, 311)
**CSF Lymphocyte** % (Median(IQR))	260	78 (46, 93)	72	99 (79, 100)	132	67 (37, 94)	464	78 (47, 96)
**CSF Protein (g/l)** (Median(IQR))	303	0.98 (0.55, 1.85)	180	0.38 (0.22, 1.42)	132	1.38 (0.47, 2.83)	615	0.87 (0.38, 1.93)
**CSF:blood glucose ratio** (Median(IQR))	303	0.48 (0.32, 0.62)	86	0.64 (0.53, 0.70)	132	0.30 (0.14, 0.50)	521	0.48 (0.27, 0.63)
**CSF volume for mycobacterial tests (mls)** (Median(IQR))	302	4.5 (4.0, 5.0)	180	12.5 (8.5, 12.5)	131	7.5 (7.0, 8.5)	613	6.0 (4.5, 8.5)

a Total no reflects the total number of observations available for the corresponding variable. The clinical diagnostic score for TBM (final diagnosis) could not be calculated in 2 individuals. GCS; Glasgow Coma Score, TBM; tuberculous meningitis, MRC Grade; denotes modified British Medical Research Council criteria. Grade 1 indicates a Glasgow coma score of 15 with no neurological signs, grade 2 a score of 11 to 14 (or 15 with focal neurological signs), and grade 3 a score of 10 or less, CSF; cerebrospinal fluid, WCC; white cell count, IQR; interquartile range.

b TBM grade shown for 380/381 patients with definite probable or possible TBM. TBM grade data missing for one patient enrolled in Indonesia.

c Chest X-ray recorded as ‘other’ if chest X-ray was performed and the appearances were not of pulmonary or miliary TB, nor were they normal.

### Heterogeneity between sites

Marked heterogeneity was noted between sites. For patients enrolled in South Africa, a greater proportion were not-TBM (59.3%, vs. 38.2% and 8.3%), had HIV co-infection (83%, vs. 7.6% and 15.2%), and had TBM grade 1 disease (48.6%, vs. 34.2% and 5.0%) compared with Vietnam and Indonesia, respectively. Patients enrolled in Indonesia were younger (median age 30 years, vs. 40 and 39 years), and more likely to have pulmonary TB (53.0% vs. 14.8% and 31.3%) compared with Vietnam and South Africa, respectively.

### Diagnostic performance

According to the clinical gold standard, 381/616 (61.9%) patients had a clinical diagnosis of TBM (definite, probable, or possible) overall; 187/303 (61.7%), 74/182 (40.7%) and 120/131 (91.6%) in Vietnam, South Africa and Indonesia, respectively. Test comparisons against the clinical gold standard are shown in Table 2. For all sites combined, the sensitivities of CZN and MZN with cytospin were 33.9% (129/380; 95% CI 29.4–38.8%) and 34.5% (129/374; 95% CI 29.9–39.4%) respectively (*p* = 1.0). Specificities of both tests were 100%. CZN was significantly more sensitive than MZN without cytospin (33.9% vs. 30.9%, *p* = 0.025). The sensitivities of CZN, MZN with cytospin and MZN without cytospin in HIV co-infected individuals were 19.8%, 15.7% and 16.5%, respectively. The sensitivities of culture and Xpert were 31.8% (119/374; 95% CI 27.3–36.7%) and 25.1% (95/379; 95% CI 21.0–29.7%), respectively.

**Table 2:** The diagnostic performance of CZN, MZN with cytospin, MZN without cytospin, culture and Xpert against clinical TBM diagnosis (definite, probable and possible TBM) as a gold standard.

	CZN	MZN with cytospin	MZN without cytospin	culture	Xpert
	(*N* = 612)	(*N* = 605)	(*N* = 604)	(*N* = 602)	(*N* = 610)
Positive tests in TBM	129/380	129/374	116/375	119/374	95/379
Sensitivity (%)	33.9	34.5	30.9	31.8	25.1
(95% CI)	(29.4–38.8%)	(29.9–39.4%)	(26.5–35.8%)	(27.3–36.7%)	(21.0–29.7%)
Specificity (%)	100	100	99.6	100	100
(95% CI)		(98.4–100%)	(97.6–100%)		
PPV (%)	100	100	99.1	100	100
(95% CI)	(97.1–100%)	(97.1–100%)	(95.3–100%)	(96.9–100%)	(96.1–100%)
NPV (%)	48	48.5	46.8	47.2	44.9
(95% CI)	(43.6–52.5%)	(44.1–53.0%)	(42.4–51.3%)	(42.8–51.7%)	(40.6–49.2%)

CZN; conventional Ziehl–Neelsen smear, MZN; modified Ziehl–Neelsen smear, culture; mycobacterial culture by MGIT (Becton Dickinson, USA) (Vietnam and South Africa) or MODS (Indonesia), Xpert; Xpert MTB/RIF (Cepheid, USA), PPV; positive predictive value, NPV; negative predictive value, CI; confidence interval. No CIs are shown for CZN, culture and Xpert specificity values. These tests are included in the reference gold standard. A positive result will always occur in a definite TBM case; therefore no level of error can be associated with the specificity value. Data shown for 613 patients (5 patients excluded, due to missing clinical diagnostic score for TBM (*n* = 2), or due to missing baseline lumbar puncture (*n* = 3)).

Test diagnostic performances of CZN and MZN compared to the gold standard stratified by site are shown in Table 3. The sensitivities of CZN and MZN with cytospin stratified by site were 47.3% vs. 49.7% (Vietnam), 12.2% vs. 9.6% (South Africa) and 26.7% vs. 26.7% (Indonesia) respectively. Both CZN and MZN with cytospin had 100% specificity at all three study sites. The sensitivities of CZN and MZN with cytospin compared against definite plus probable TBM, and definite TBM alone, are shown in supplementary Tables 3 and 4, respectively. Using positive culture as a reference, sensitivities for TBM diagnosis of CZN and MZN with cytospin were 66.4% and 67.5%, respectively (*p* = 1.0 for the difference between tests), and Xpert sensitivity against culture was 72.3%. Culture and Xpert were more sensitive than CZN and MZN with cytospin with the exception of Vietnam where microscopy was more sensitive than culture or Xpert, as previously described.^4^ The sensitivities of CZN, MZN with cytospin, MZN without cytospin, and culture were statistically superior to Xpert in the overall analysis (sensitivities; 33.9% vs. 25.1% *p* < 0.001, 34.5% vs. 25.1% *p* < 0.001, 30.9% vs. 25.1% *p* = 0.032, 31.8% vs. 25.1% *p* < 0.001, respectively).

**Table 3:** The sensitivity and specificity (with 95% CIs) for CZN, MZN with cytospin, MZN without cytospin, culture, and Xpert compared to clinical diagnosis (definite, probable and possible TBM) as gold standard stratified by site.

		CZN	MZN with cytospin	MZN without cytospin	Culture	Xpert
Positive tests	Vietnam	88/186	90/181	82/181	47/182	37/186
	South Africa	9/74	7/73	7/74	20/72	13/73
	Indonesia	32 / 120	32/120	27/120	52/120	45/120
Sensitivity (95% CI)	Vietnam	47.3%	49.7%	45.3%	25.8%	19.9%
		(40.3–54.5%)	(42.5–56.9%)	(38.2–52.6%)	(20.0–32.6%)	(14.8–26.2%)
	South Africa	12.2 %	9.6 %	9.5%	27.8 %	17.8 %
		(6.5–21.5%)	(4.7–18.5%)	(4.7–18.3%)	(18.8–39.0%)	(10.7–28.1%)
	Indonesia	26.7%	26.7%	22.5%	43.3%	37.5%
		(19.6–35.2%)	(19.6–35.2%)	(15.9–30.8%)	(34.8–52.3%)	(29.4–46.4%)
Specificity (95% CI)	Vietnam	100%	100%	99.1%	100%	100%
			(96.8–100%)	(95.2–100%)		
		100%	100%	100%	100%	100%
			(96.5–100%)	(96.5–100%)		
	South Africa					
	Indonesia	100%	100%	100%	100%	100%
			(74–100%)	(74.1–100%)		

CZN; conventional Ziehl–Neelsen smear, MZN; modified Ziehl–Neelsen smear, culture; mycobacterial culture by MGIT (Becton Dickinson, USA) (Vietnam and South Africa) or MODS (Indonesia), Xpert; Xpert MTB/RIF (Cepheid, USA), CI; confidence interval. No CIs are shown for CZN, culture and Xpert specificity values. These tests are included in the reference gold standard. A positive result will always occur in a definite TBM case; therefore no level of error can be associated with the specificity value. Data shown for 613 patients (5 patients excluded, due to missing clinical diagnostic score for TBM (*n* = 2), or due to missing baseline lumbar puncture (*n* = 3)).

### Positive mycobacterial tests

**Fig. 2:**
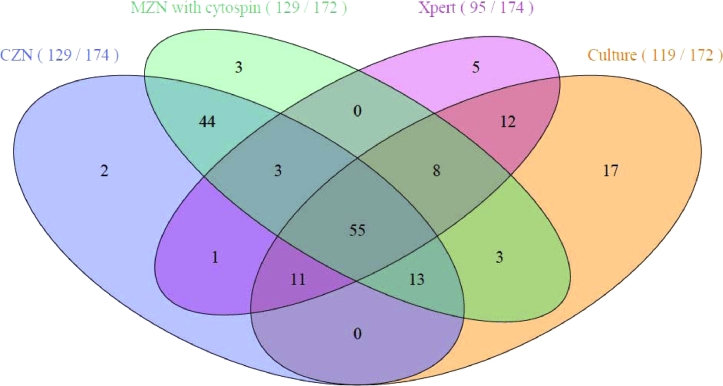
Venn diagram of mycobacterial tests (conventional ZN, modified ZN with cytospin, Xpert and culture). CZN; conventional Ziehl–Neelsen smear, MZN; modified Ziehl–Neelsen smear, Xpert; Xpert MTB/RIF (Cepheid), culture; mycobacterial culture by MGIT (Becton Dickinson, USA) (Vietnam and South Africa) or MODS (Indonesia). For each test, the number of positive results is shown divided by the number of tests performed. Test denominators (CZN 174, MZN with cytospin 172, Xpert 174, culture 172) refer to the number of definite cases of TBM for which each test was performed.

Fig. 2 shows mycobacterial tests positive by CZN, MZN with cytospin, Xpert and culture (in 174 definite TBM cases, plus 3 additional cases positive by MZN with cytospin alone (all possible TBM). A total of 66 cases were positive by CZN, Xpert and culture. Two additional cases were detected by CZN alone, 3 by MZN with cytospin alone, 2 by MZN without cytospin alone, 5 by Xpert alone, and 17 by culture alone. Time to positive culture, defined as the number of days since LP was performed to reporting of positive culture, was 16 days in Vietnam and 20.5 days in South Africa (*p* = 0.033). In Indonesia, where MODS culture was performed instead of MGIT, time to positivity was 18.5 days on a weekly observation. MODS showed a comparable sensitivity to MGIT, but shorter time to positivity in a previous study of TBM in Vietnam.^9^

### Comparison of the modified ZN method with and without cytospin

The sensitivities of MZN with cytospin and MZN without cytospin against clinical diagnosis were 34.5% (129/374; 95% CI 29.9–39.4%) and 30.9% (116/375; 95% CI 26.5–35.8%) respectively, with cytospin preparation significantly increasing sensitivity (*p* = 0.016, Table 2). Specificities were 100% (98.4–100%) and 99.6% (97.6–100%), respectively.

### Predictors of microbiologically confirmed TBM

We performed univariate and multivariate analyses to assess parameters that positively correlated with microbiological diagnosis (defined as positive CZN, Xpert or culture) of TBM. All cases of definite, probable or possible TBM were included in the analysis (*n* = 381). Univariate logistic regression results are described in supplementary material 2. In the multivariate analysis the following factors were independently and positively associated with microbiological confirmation of TBM: higher CSF volume, higher CSF lactate. The following factors were associated negatively with microbiological confirmation of TBM (i.e. factors associated with less likely microbiological confirmation): increasing age, TBM grade 3 (compared to grade 1), location of Indonesia (compared to Vietnam), and an increased CSF:blood glucose ratio (Table 4).

**Table 4:** A univariate and multivariate analysis of factors associated with microbiological confirmation of TBM.

Factor	Univariate model			Multivariate model		
	Odds ratio	95% CI	*P* value	Odds ratio	95% CI	*P* value
Age (years)	0.984	0.970–0.998	0.028	0.968	0.946–0.990	0.004
Female sex	0.72	0.48–1.08	0.115	0.58	0.33–1.03	0.060
TBM MRC Grade			0.101			0.054
TBM MRC Grade 2 (compared to Grade 1)	1.61	1.00–2.58	0.049	0.94	0.46–1.92	0.870
TBM MRC Grade 3 (compared to Grade 1)	1.07	0.55–2.10	0.840	0.32	0.11–0.94	0.037
HIV negative	2.49	1.47–4.22	<0.001	2.07	0.71–5.99	0.180
Not received BCG	1.78	1.08–2.94	0.022	1.67	0.77–3.63	0.190
Location			0.011			0.032
Located in Indonesia (compared to Vietnam)	0.98	0.62–1.55	0.920	0.19	0.06–0.65	0.007
Located in South Africa (compared to Vietnam)	0.43	0.24–0.76	0.004	0.22	0.03–1.44	0.110
CSF volume (ml)	0.95	0.88–1.02	0.138	1.30	1.04–1.63	0.021
CSF appearance			0.223			0.139
Cloudy (compared to clear)	1.35	0.43–4.26	0.610	0.25	0.05–1.19	0.080
Mild cloudy/opalescent (compared to clear)	1.66	0.92–3.00	0.090	0.56	0.21–1.45	0.230
CSF colour			<0.001			0.765
Bloody (compared to colourless)	1.39	0.41–4.74	0.590	0.76	0.15–3.84	0.740
White (compared to colourless)	4.93	0.96–25.41	0.060	1.49	0.18–12.22	0.710
Yellow (compared to colourless)	3.26	1.92–5.55	<0.001	0.68	0.28–1.62	0.380
CSF neutrophil percentage	1.015	1.007–1.024	<0.001	1.007	0.996–1.018	0.240
CSF:blood glucose ratio	0.002	0.00–0.07	<0.001	0.01	0–0.12	<0.001
Log2 CSF Lactate	4.70	2.95–7.50	<0.001	3.37	1.25–9.12	0.012
Log10 CSF Protein	8.99	5.06–15.98	<0.001	1.52	0.53–4.35	0.430
Log2 symptom duration (days)	1.05	0.88–1.24	0.550	1.04	0.80–1.35	0.760

Odds ratios are displayed for an increase in 1 unit of each variable. For categorical variables with more than 2 levels (location, TBM Grade, CSF appearance, CSF colour) the multivariate Wald test was used. MRC grade; denotes modified British Medical Research Council criteria. Grade 1 indicates a Glasgow coma score of 15 with no neurological signs, grade 2 a score of 11–14 (or 15 with focal neurological signs), and grade 3 a score of 10 or less, BCG; Bacillus Calmette–Guerin vaccine, CSF; cerebrospinal fluid, CI; confidence interval. Data shown for 613 patients (5 patients excluded, due to missing clinical diagnostic score for TBM (*n* = 2), or due to missing baseline lumbar puncture (*n* = 3)).

## Discussion

The diagnosis of TBM is very challenging. Early recognition and appropriate antitubercular therapy improve chances of survival. However, current diagnostic tests lack sufficient sensitivity. CSF smear, Xpert and culture are often unable to detect MTB found in the paucibacillary CSF of individuals with TBM. TBM cannot be excluded with a negative mycobacterial test. CSF smear microscopy performance is highly variable, and requires large volume CSF sampling and experienced microscopists. Xpert is useful when positive, gives rapid results and identifies rifampicin resistance. Mycobacterial culture and susceptibility results refine drug therapy but cannot affect early therapy. Additional tests that offer early diagnosis with high sensitivity are urgently required for TBM.

We found that the performance of MZN with cytospin was similar to CZN (34.5% vs. 33.9%, *p* = 1.0). We were unable to reproduce the higher sensitivities associated with MZN reported from China,^5^, ^6^ which may be due to lower volumes of CSF used for CZN in their studies. In addition, the specificity of MZN was lower in the Chinese studies than in our study (85.0% and 71.4%, vs. 100%, respectively).^6^, ^7^ There is no confirmed explanation for this discrepancy in specificity, but it could be due to misidentification of AFB.

MZN with cytospin adds no additional diagnostic advantage if CZN is also performed. In our overall analysis the sensitivities of CZN, MZN with cytospin, and culture were all statistically superior to Xpert. Overall superiority of smear over Xpert was due to the high smear sensitivity seen in Vietnam. Both CZN and MZN with cytospin were more sensitive than Xpert in Vietnam (47.3% and 49.7% respectively, vs. 19.9%), however in South Africa and Indonesia both CZN and MZN with cytospin were less sensitive than Xpert. The purpose of MZN was to permeabilise cells (Triton step) and allow access of carbolfuchsin to stain intracellular mycobacteria. By omitting the cytospin step, the technique would be achievable in resource poor settings. However, MZN without cytospin was inferior to CZN. The addition of the cytospin step during MZN staining increased MTB detection, but did not exceed the performance of CZN.

In 2004 Thwaites et al. demonstrated that volume of CSF tested is independently associated with microbiological confirmation of TBM.^10^ This was also recently found in a study of 573 CSF samples for which volume was recorded in Indonesia.^11^ Our multivariate analysis results confirm this important association (Table 4). In addition to CSF volume, our multivariate analysis corroborates associations with CSF:blood glucose ratio, and higher CSF lactate, with microbiologically confirmed TBM. Both indicators correlate with more severe disease, possibly due to higher mycobacterial loads, resulting in increased detection.

The strength of our study is that it was a large, prospective, international multicentre study, demonstrating that MZN with cytospin is not a more sensitive diagnostic test than CZN for TBM, across all sites combined, or at any site analysed individually. The results contradict earlier reports.^5^, ^6^, ^7^ The heterogeneity between patient populations across the study sites enhances the generalisability of the results, although it makes the interpretation of the overall analysis more challenging. For example, patients from South Africa had a markedly higher rate of HIV co-infection, and the univariate (but not multivariate) association between HIV negative status and microbiological confirmation of TBM may be explained by 151/194 (77.8%) of all study patients with HIV enrolling in South Africa. Patients in South Africa also had the lowest median CSF cell count and CSF protein concentration, and the highest CSF:blood glucose ratio, which may be caused by HIV-coinfection or suggest less severe disease. This could also reflect a lower threshold for performing lumbar puncture at this site, where TB prevalence is high, HIV co-infection is common, and lumbar puncture is readily available, resulting in earlier TBM diagnosis. Alternatively or additionally, in some “possible” cases mild CSF changes due to HIV-infection itself may have wrongly been attributed to meningitis. Slight differences in slide reading may also have accounted for some of the differences in performance between sites. For example, the high sensitivity of microscopy in Vietnam may be because all the fields were examined, rather than just the first 100. Marked heterogeneity in history of previous TB was also seen between sites, with a much lower rate in Vietnam. These population differences may affect the average CSF bacillary load, and mycobacterial test sensitivity will vary in tandem. In addition, microscopist experience varied between sites, and between individuals within a site, which may also have influenced performance.

Another potential limitation of this study is incorporation bias; multiple mycobacterial tests were compared against a clinical case definition that CZN, culture and Xpert were also part of, yet this was necessary as no single diagnostic test is sufficiently sensitivity to be a gold standard. False positives for CZN, culture and Xpert would not have been detected in this analysis as these cases would have been classified as definite TBM on the basis of this positive test alone. Modified ZN smears, with or without cytospin, were not included in the clinical case definition, as these were the tests under investigation.

Currently, physicians worldwide rely on CSF ZN staining, Xpert and culture for the microbiological diagnosis of TBM. We found that modifications to the ZN stain, that enhanced the performance of CSF microscopy in previous studies,^5^, ^6^ did not improve upon the CZN stain for TBM diagnosis. Cytospin preparation of slides may enhance the performance of microscopy, but the cost of the centrifuge would need to be balanced against the small absolute increase in sensitivity (∼5%). The performance of meticulous microscopy is equivalent or superior to Xpert; but Xpert is less user dependent than microscopy and offers the major advantage of rifampicin susceptibility prediction. Xpert Ultra promises substantially enhanced sensitivity,^12^ but its impact needs confirmation in larger prospective studies. New high sensitivity diagnostic tests are urgently required to stop patients dying from TBM. Until they arrive however, we must make best use of the tests we have, and optimise their performance by taking and testing large volumes of CSF.

## Appendix Supplementary materials

Image, application 1
