# Supplementary material for: Improving the microbiological diagnosis of tuberculous meningitis: A prospective, international, multicentre comparison of conventional and modified Ziehl–Neelsen stain, GeneXpert, and culture of cerebrospinal fluid
Source: J Infect. 2018 Dec;77(6):509–15. doi: 10.1016/j.jinf.2018.09.003 (PMC6293313; doi:10.1016/j.jinf.2018.09.003)
Supplement: Supplementary file 1 [file mmc1.zip › mmc1.docx]

## Supplementary appendix

**Supplementary table 1: Diagnostic criteria for TBM according to the consensus case definition [8].**

|  |  | Diagnostic score | |
| --- | --- | --- | --- |
| **Clinical criteria** | (Maximum category score=6) | | |
|  | Symptom duration of >5 days | 4 | |
|  | Systemic symptoms suggestive of tuberculosis (one or more of the following): weight loss (or poor weight gain in children), night sweats, or persistent cough for >2 weeks | 2 | |
|  | History of recent (within past year) close contact with an individual with pulmonary tuberculosis or a positive TST or IGRA (only in children <10 years of age) | 2 | |
|  | Focal neurological deficit (excluding cranial nerve palsies) | 1 | |
|  | Cranial nerve palsy | 1 | |
|  | Altered consciousness | 1 | |
| **CSF criteria** | (Maximum category score=4) | | |
|  | Clear appearance | 1 | |
|  | Cells: 10-500 per μl | 1 | |
|  | Lymphocytic predominance (>50%) | 1 | |
|  | Protein concentration >1 g/L | 1 | |
|  | CSF to plasma glucose ratio of less than 50% or an absolute CSF glucose concentration less than 2.2mmol/L | 1 | |
| **Cerebral imaging criteria** | (Maximum category score=6) | | |
|  | Hydrocephalus | 1 | |
|  | Basal meningeal enhancement | 2 | |
|  | Tuberculoma | 2 | |
|  | Infarct | 1 | |
|  | Pre-contrast basal hyperdensity | 2 | |
| **Evidence of tuberculosis elsewhere** | (Maximum category score=4) | | |
|  | Chest radiograph suggestive of active tuberculosis: signs of tuberculosis=2; miliary tuberculosis=4 | 2 or 4 | |
|  | CT/ MRI/ ultrasound evidence for tuberculosis outside the CNS | 2 | |
|  | AFB identified or *Mycobacterium tuberculosis* cultured from another source-i.e., sputum, lymph node, gastric washing, urine, blood culture | 4 | |
|  | Positive commercial *M. tuberculosis* NAAT from extra-neural specimen | 4 | |
| **Diagnostic criteria based on total score:**  Not TBM: score < 6, or alternative diagnosis established without a definitive diagnosis of TBM or other convincing signs of dual disease  Possible TBM: score 6-9 (if no brain imaging) or 6-11 (if brain imaging)  Probable TBM: score >9 (if no brain imaging) or >11 (if brain imaging)- at least 2 points should either come from cerebrospinal fluid (CSF) or brain imaging criteria  Definite TBM: acid-fast bacilli seen in CSF or *M. tuberculosis* cultured or detected by commercial NAAT in CSF | | |  |

**Supplementary table 2: Baseline characteristics by TBM diagnosis**

|  | Definite TBM  (N = 174) | Probable TBM (N = 50) | Possible TBM  (N = 157) | Not TBM  (N = 235) | Total  (N = 616) |
| --- | --- | --- | --- | --- | --- |
| **Age (years)** (Median(IQR)) | 35 (26, 45) | 40 (30, 49) | 37 (27, 51) | 39 (30, 53) | 37 (28, 50) |
| **Sex (male)** (No(%)) | 104 (59.8%) | 30 (60.0%) | 77 (49.0%) | 141 (60.0%) | 352 (57.1%) |
| **Study site** (No(%))  Vietnam  South Africa  Indonesia | 93 (53.4%)  22 (12.6%)  59 (33.9%) | 11 (22.0%)  8 (16.0%)  31 (62.0%) | 83 (52.9%)  44 (28.0%)  30 (19.1%) | 116 (49.4%)  108 (46.0%)  11 (4.7%) | 303 (49.2%)  182 (29.5%)  131 (21.3%) |
| **HIV status** (No(%))  Positive  Negative  Unknown | 30 (17.2%)  142 (81.6%)  2 (1.1%) | 16 (32.0%)  32 (64.0%)  2 (4.0%) | 45 (28.7%)  76 (48.4%)  36 (22.9%) | 103 (43.8%)  76 (32.3%)  56 (23.8%) | 194 (31.5%)  326 (52.9%)  96 (15.6%) |
| **Duration of illness** (days) (Median(IQR)) | 11 (7, 20) | 14 (8, 21) | 10 (6, 18) | 5 (3, 14) | 9 (5, 16) |
| **Known history of TB** (No(%))  Yes  No  Unknown | 20 (11.5%)  139 (79.9%)  15 (8.6%) | 21 (42.0%)  26 (52.0%)  3 (6.0%) | 34 (21.7%)  110 (70.1%)  13 (8.3%) | 68 (28.9%)  151 (64.3%)  16 (6.8%) | 143 (23.2%)  426 (69.2%)  47 (7.6%) |
| **TBM MRC Grade^1^**  (No(%))  1  2  3  Unknown | 41 (23.6%)  111 (63.8%)  22 (12.6%)  0 | 9 (18.0%)  34 (68.0%)  7 (14.0%)  0 | 56 (35.7%)  75 (47.7%)  25 (15.9%)  1 (0.6%) |  | 106 (27.8%)  220 (57.7%)  54 (14.2%)  1 (0.0%) |
| **Cranial Nerve Palsy**  (No(%))   Yes  No | 62 (35.6%)  112 (64.4%) | 21 (42.0%)  29 (58.0%) | 24 (15.3%)  133 (84.7%) | 26 (11.1%)  209 (88.9%) | 133 (21.6%)  483 (78.4%) |
| **Hemiplegia**  (No(%))  Yes  No  Unknown | 25 (14.4%)  146 (83.9%)  3 (1.7%) | 14 (28.0%)  36 (72.0%)  0 | 16 (10.2%)  139 (88.5%)  2 (1.3%) | 19 (8.1%)  215 (91.5%)  1 (0.4%) | 74 (12.0%)  536 (87.0%)  6 (1.0%) |
| **Paraplegia**  (No(%))  Yes  No  Unknown | 11 (6.3%)  161 (92.5%)  2 (1.1%) | 5 (10.0%)  45 (90.0%)  0 | 9 (5.7%)  146 (93.0%)  2 (1.3%) | 3 (1.3%)  231 (98.3%)  1 (0.4%) | 28 (4.6%)  583 (94.6%)  5 (0.8%) |
| **Tetraplegia**  (No(%))  Yes  No  Missing | 2 (1.1%)  170 (97.7%)  2 (1.1%) | 0  50 (100.0%)  0 | 3 (1.9%)  151 (96.1%)  3 (1.9%) | 1 (0.4%)  230 (97.9%)  4 91.7%) | 6 (1.0%)  601 (97.6%)  9 (1.5%) |
| **Seizures**  (No(%))  Yes  No  Unknown | 7 (4.0%)  164 (94.3%)  3 (1.7%) | 6 (12.0%)  44 (88.0%)  0 | 23 (14.6%)  128 (81.5%)  6 (3.8%) | 38 (16.2%)  192 (81.7%)  5 (2.1%) | 74 (12.0%)  528 (85.7%)  14 (2.3%) |
| **GCS^2^** (Median(IQR)) | 14 (12, 15) | 14 (12, 15) | 14 (12, 15) | 14 (13, 15) | 14 (12, 15) |
| **Chest X-ray^3^**  (No(%))  Pulmonary TB  Miliary TB  Normal  Other | 79 (46.2%)  29 (17.0%)  51 (29.8%)  12 (7.0%) | 35 (71.4%)  4 (8.2%)  9 (18.4%)  1 (2.0%) | 34 (22.7%)  2 (1.3%)  86 (57.3%)  28 (18.7%) | 24 (11.7%)  1 (0.5%)  135 (65.9%)  45 (22.0%) | 172 (27.9%)  36 (5.8%)  281 (45.6%)  86 (14.0%) |
| **CSF WCC (per mm^3^)^4^**  (Median(IQR)) | 279 (164, 476) | 28 (3, 126) | 21 (1, 182) | 17 (1, 185) | 80 (3, 311) |
| **CSF Lymphocyte %^5^**  (Median(IQR)) | 72 (40, 90) | 88 (59, 99) | 89 (58, 98) | 80 (46, 96) | 78 (47, 96) |
| **CSF Protein (g)^6^**  (Median(IQR)) | 1.80 (1.20, 2.84) | 1.15 (0.41, 2.83) | 0.54 (0.27, 1.36) | 0.59 (0.32, 0.95) | 0.87 (0.38, 1.92) |
| **CSF:blood glucose ratio^7^**  (Median(IQR)) | 0.25 (0.14, 0.37) | 0.41 (0.27, 0.50) | 0.56 (0.43, 0.66) | 0.60 (0.46, 0.72) | 0.48 (0.27, 0.63) |
| **CSF Volume for mycobacterial tests (mls)^8^**  (Median(IQR)) | 5.5 (5.0, 7.7) | 6.8 (5.5, 8.0) | 5.0 (4.5, 8.5) | 6.5 (4.5, 12.5) | 6.0 (4.5, 8.5) |

Table legend

**^1^**TBM grade data shown for only those patients with definite probable or possible TBM. **^2^**GCS data missing for 1 patient in the possible TBM group. **^3^**No chest X-ray was performed or data missing for 41 patients, 3 in the definite TBM group, 1 in the probable TBM group, 7 in the possible TBM group, and 30 in the not-TBM group. **^4^**CSF WCC data not available for 5 patients, 1 in the possible TBM group and 4 in the not-TBM group. **^5^**CSF Lymphocyte % data missing for 153 patients, 2 in the definite TBM group, 3 in the probable TBM group, and 46 in the possible TBM group. CSF Lymphocyte data is not available for 102 patients in the not-TBM group, however the lymphocyte proportion could not be determined where no cells were seen in the CSF. **^6^**CSF Protein data missing for 2 patients, 1 in the definite TBM group and 1 in the not-TBM group. ^7^CSF:Blood glucose ratio data missing for 96 patients, 19 in the definite TBM group, 6 in the probable TBM group, 22 in the possible TBM group and 49 in the not-TBM group. ^8^CSF Volume data missing for 3 patients, 1 in the possible TBM group and 2 in the not-TBM group. MRC grade; denotes modified British Medical Research Council criteria. Grade 1 indicates a Glasgow coma score of 15 with no neurological signs, grade 2 a score of 11 to 14 (or 15 with focal neurological signs), and grade 3 a score of 10 or less, GCS; Glasgow coma score, CSF; cerebrospinal fluid, WCC; white cell count, IQR; interquartile range

**Supplementary table 3: The diagnostic performance of CZN, MZN with cytospin, culture and Xpert against clinical TBM diagnosis (definite and probable TBM) as a gold standard**

|  | CZN  (N = 612) | MZN with cytospin (N = 605) | culture  (N = 602) | Xpert  (N = 610) |
| --- | --- | --- | --- | --- |
| Positive tests  in TBM | 129 / 224 | 126 / 222 | 119 / 221 | 95 / 223 |
| Sensitivity  (95% CI) | 57.6%  (51.0 - 63.9%) | 56.8%  (50.2 - 63.1%) | 53.8%  (47.3 - 60.3%) | 42.6%  (36.3 - 49.2%) |
| Specificity  (95% CI) | 100% | 99.2%  (97.7 - 99.7%) | 100% | 100% |
| PPV  (95% CI) | 100%  (97.1 - 100%) | 97.7%  (3.4 - 99.2%) | 100%  (96.9 - 100%) | 100%  (96.1 - 100%) |
| NPV  (95% CI) | 80.3%  (76.6 - 83.6%) | 79.8%  (76 - 83.2%) | 78.9%  (75.0 - 82.3%) | 75.1%  (71.2 - 78.7%) |

Table legend

CZN; conventional Ziehl-Neelsen smear, MZN; modified Ziehl-Neelsen smear, culture; mycobacterial culture by MGIT (Becton Dickinson, USA) (Vietnam and South Africa) or MODS (Indonesia), Xpert; Xpert MTB/RIF (Cepheid, USA), PPV; positive predictive value, NPV; negative predictive value, CI; confidence interval. No CIs are shown for CZN, culture and Xpert specificity values. These tests are included in the reference gold standard. A positive result will always occur in a definite TBM case; therefore no level of error can be associated with the specificity value.

**Supplementary table 4: The diagnostic performance of CZN, MZN with cytospin, culture and Xpert against definite TBM as a gold standard**

|  | CZN  (N = 612) | MZN with cytospin (N = 605) | culture  (N = 602) | Xpert  (N = 610) |
| --- | --- | --- | --- | --- |
| Positive tests  in TBM | 129 / 174 | 126 / 172 | 119 / 172 | 95 / 174 |
| Sensitivity  (95% CI) | 74.1%  (67.2 - 80.1%) | 73.3%  (66.2 - 79.3%) | 69.2%  (61.9 - 75.6%) | 54.6%  (47.2 - 61.8%) |
| Specificity  (95% CI) | 100% | 99.3%  (98 - 99.8%) | 100% | 100% |
| PPV  (95% CI) | 100%  (97.1 - 100%) | 97.7%  (93.4 - 99.2%) | 100%  (96.9 - 100%) | 100%  (96.1 - 100%) |
| NPV  (95% CI) | 90.7%  (87.8 - 93%) | 90.3%  (87.3 - 92.7%) | 89%  (85.9 - 91.5%) | 84.7%  (81.3 - 87.5%) |

Table legend

CZN; conventional Ziehl-Neelsen smear, MZN; modified Ziehl-Neelsen smear, culture; mycobacterial culture by MGIT (Becton Dickinson, USA) (Vietnam and South Africa) or MODS (Indonesia), Xpert; Xpert MTB/RIF (Cepheid, USA), PPV; positive predictive value, NPV; negative predictive value, CI; confidence interval. No CIs are shown for CZN, culture and Xpert specificity values. These tests are included in the reference gold standard. A positive result will always occur in a definite TBM case; therefore no level of error can be associated with the specificity value.

**Supplementary materials:**

1. Method for MZN with cytospin preparation

MZN with cytospin was performed as follows; 0.5 mls of CSF was loaded into a cytospin chamber with poly-l-lysine-coated slides and centrifuged at 100 g for 10 minutes. The slide was fixed with 4% paraformaldehyde for 15 minutes at room temperature then permeabilised with 0.3% Triton X-100 for 30 minutes, before staining with carbolfuchsin containing 0.3% Triton X-100 and counterstained with methylene blue. In Vietnam and Indonesia the slides were examined under a light-microscope for acid-fast bacilli (AFB) by two experienced laboratory technicians. Positive slides were confirmed between the technicians. In South Africa one microbiologist reviewed the slides. At least one hundred fields of each slide were examined. For the modified stain with omission of the cytospin step (MZN without cytospin), 100 µL aliquot of the deposit from the centrifuged whole CSF was stained by modified ZN stain with omission of the cytospin step. The 100 µL aliquot was heat fixed on a poly-l-lysine slide. Triton X-100 was added for 30 minutes to the slide, before staining with carbolfuchsin containing 0.3% Triton X-100 and counterstained with methylene blue.

2. Univariate analysis

Univariate and multivariate analyses were performed on the following parameters; age (years), sex, TBM grades 2 and 3 (compared to grade 1), HIV status, Bacillus Calmette Guerin (BCG) vaccine status, study site, CSF volume (mls), CSF appearance, CSF colour, CSF neutrophil percentage, CSF : blood glucose ratio, CSF lactate, CSF protein and duration of symptoms.

Based on the univariate logistic regression, the following factors were positively associated with microbiological confirmation of TBM: HIV negative status, not having received BCG vaccine, yellow CSF compared to colourless, increased CSF neutrophil percentage, increased CSF lactate and increased CSF protein. The following factors were associated negatively with microbiological confirmation of TBM (i.e. they were associated with less likely microbiological confirmation): increasing age, location of South Africa (compared to Vietnam), and an increased CSF: blood glucose ratio.
